# Intermittent theta-burst stimulation to the right dorsolateral prefrontal cortex may increase potentiated startle in healthy individuals

**DOI:** 10.1038/s41386-024-01871-w

**Published:** 2024-05-13

**Authors:** Marta Teferi, Hannah Gura, Milan Patel, Abigail Casalvera, Kevin G. Lynch, Walid Makhoul, Zhi-De Deng, Desmond J. Oathes, Yvette I. Sheline, Nicholas L. Balderston

**Affiliations:** 1 Center for Neuromodulation in Depression and Stress Department of Psychiatry University of Pennsylvania, Philadelphia, PA USA; 2 Neuroscience Graduate Group Perelman School of Medicine University of Pennsylvania, Philadelphia, PA USA; 3 Department of Psychiatry University of Pennsylvania, Philadelphia, PA USA; 4 Noninvasive Neuromodulation Unit Experimental Therapeutics and Pathophysiology Branch National Institute of Mental Health National Institutes of Health Bethesda, Bethesda, MD USA; 5 Center for Brain Imaging and Stimulation Department of Psychiatry University of Pennsylvania, Philadelphia, PA USA; 6 Penn Brain Science, Translation, Innovation, and Modulation Center University of Pennsylvania, Philadelphia, PA USA

**Keywords:** Human behaviour, Anxiety

## Abstract

Repetitive transcranial magnetic stimulation (rTMS) treatment protocols targeting the right dlPFC have been effective in reducing anxiety symptoms comorbid with depression. However, the mechanism behind these effects is unclear. Further, it is unclear whether these results generalize to non-depressed individuals. We conducted a series of studies aimed at understanding the link between anxiety potentiated startle and the right dlPFC, following a previous study suggesting that continuous theta burst stimulation (cTBS) to the right dlPFC can make people more anxious. Based on these results we hypothesized that intermittent TBS (iTBS), which is thought to have opposing effects on plasticity, may reduce anxiety when targeted at the same right dlPFC region. In this double-blinded, cross-over design, 28 healthy subjects underwent 12 study visits over a 4-week period. During each of their 2 stimulation weeks, they received four 600 pulse iTBS sessions (2/day), with a post-stimulation testing session occurring 24 h following the final iTBS session. One week they received active stimulation, one week they received sham. Stimulation weeks were separated by a 1-week washout period and the order of active/sham delivery was counterbalanced across subjects. During the testing session, we induced anxiety using the threat of unpredictable shock and measured anxiety potentiated startle. Contrary to our initial hypothesis, subjects showed increased startle reactivity following active compared to sham stimulation. These results replicate work from our two previous trials suggesting that TMS to the right dlPFC increases anxiety potentiated startle, independent of both the pattern of stimulation and the timing of the post stimulation measure. Although these results confirm a mechanistic link between right dlPFC excitability and startle, capitalizing upon this link for the benefit of patients will require future exploration.

## Introduction

Anxiety disorders are among the most prevalent types of psychiatric conditions, with approximately twenty percent of individuals in the United States meeting the criteria in a year [1]. Those grappling with anxiety disorders often encounter challenges in maintaining focus, concentration, and managing their attention, which can significantly impact everyday functioning [2]. Although these cognitive impairments are widespread, there is still a lack of understanding of the underlying mechanisms responsible for these deficits. A crucial aspect of understanding the anxiety/cognition interaction lies in grasping how regions responsible for cognitive control in the prefrontal cortex contribute to the expression and regulation of anxiety. The dorsolateral prefrontal cortex (dlPFC) is recognized for its significance in these interactions, playing an active role in working memory [3–6], and emotional regulation [7–9]. However, it remains uncertain whether the dlPFC’s involvement facilitates the manifestation or the regulation of anxiety.

Neuroimaging data show that the right dlPFC may play a pivotal role in the regulation of anxiety [7–9]. Our research has demonstrated several key findings in this regard. Firstly, we observed that the right dlPFC is activated in response to unpredictable threat, and this activation is inversely associated with the startle responses recorded outside of the scanner [10]. Secondly, we found that challenging cognitive tasks also engage the right dlPFC in response to unpredictable threat, and this activation is positively linked to cognitive task performance [11]. Furthermore, tasks that involve the right dlPFC have been shown to reduce state anxiety potentiated startle [5]. In individuals with anxiety disorders, including mixed generalized and social anxiety disorder, our studies have revealed that the right dlPFC exhibits either heightened or diminished activation compared to control groups, depending on the specific task performed [3, 12]. These findings align with previous research that has established a connection between abnormal activity in the right dlPFC and deficits in attentional control, observed in both anxiety patients and highly anxious individuals without clinical diagnoses [3, 12–21]. However, it is crucial to note that while accumulating evidence suggests a strong association between right dlPFC activity and anxiety regulation, these results are correlational.

Noninvasive neuromodulation techniques like transcranial magnetic stimulation (TMS) allow us to causally test such brain-behavior hypotheses by experimentally modulating neural activity in a target region or network [22]. Our work targeting the right dlPFC in anxiety has led to mixed results. In a previous study, we measured anxiety potentiated startle in healthy volunteers before and after continuous theta burst stimulation (cTBS), which is thought to induce long-term depression like changes in synaptic plasticity [23–28], and found that startle increased after active compared to sham cTBS [29]. Although these results are potentially consistent with hypotheses that the dlPFC mediates top-down control of anxiety [7–9], they are inconsistent with our previous work which demonstrated that stimulating the right dlPFC with (presumably) excitatory 10 Hz stimulation also increases anxiety potentiated startle [30]. Therefore, to disentangle these findings, here we measured startle before and after active or sham intermittent TBS (iTBS), which is thought to induce long-term potentiation like changes in synaptic plasticity [23–28]. Aside from the pattern of stimulation, all other experimental parameters were similar to those in our cTBS study [29]. Accordingly, we hypothesized that iTBS would have opposing effects on anxiety potentiated startle, resulting in a net decrease in startle after active compared to sham stimulation.

## Materials and methods

### Participants

Based on our previous work, we anticipated a moderate effect size (cohen’s d ~ 0.5–0.6). We set our power to detect an effect at 0.8 and used a corrected two-tailed alpha of 0.025. Accordingly, we anticipated needing 25–30 subjects to detect a difference between the active and sham conditions. We recruited 33 right-handed participants between the ages of 18–50 from the Philadelphia, PA metropolitan area between 9/2021 and 10/2022 to participate in this study. The exclusion criteria include current or past mood or anxiety disorder(s) as identified with the Structured Clinical Interview for DSM-5 research version (SCID-5-RV), MRI/TMS contraindications (claustrophobia, implanted metal, history or increased risk of seizure, epilepsy, etc.), any significant medical or neurological conditions (heart disease, respiratory illness, cardiovascular illness, neurological illness, etc.), and use of medications acting on the central nervous system. For a complete list, see www.clinicaltrial.gov (Identifier: NCT05322239).

A total of 28 participants completed the study (18 females, 10 males, mean age = 23.75 years, SD = 4.26). Five consented subjects were excluded from the final sample due to withdrawal from the study (1 due to scheduling; 2 no-shows; 1 due to headache from TMS stimulation; 1 due to feeling claustrophobic during MRI scan). All participants signed an informed consent form, and the protocol was approved by the Institutional Review Board for human subject research at the University of Pennsylvania. The authors assert that all procedures contributing to this work comply with the ethical standards of the relevant national and institutional committees on human experimentation and with the Helsinki Declaration of 1975, as revised in 2008.

### Procedure

#### General

**Fig. 1:**
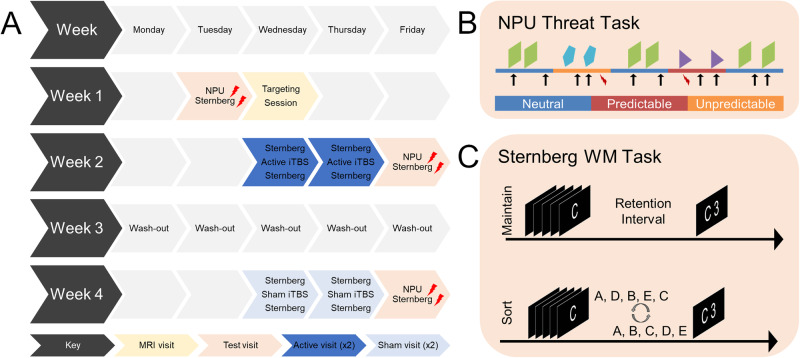
Schematic of the study designs. **A** Diagram showing protocol flow. NPU and Sternberg performance was measured before and after 4 sessions of either active or sham iTBS. **B** Diagram showing design of the NPU task. Blocks of neutral, predictable, and unpredictable conditions were presented. During the neutral periods, subjects could not receive a shock (lightning bolt). During the predictable periods, subjects could receive a shock only during the cue (shapes). During the unpredictable periods, subjects could receive a shock at any time. Blink responses to white noise probes (arrows) were measured throughout the task. **C** Diagram showing the Sternberg WM task. Subjects viewed a series of letters and either 1) maintained them in the order presented or 2) sorted them in alphabetical order. Lightning bolts indicate task that include shock presentations.

Participants completed 8 study visits over the course of 4 weeks (Fig. 1A). During Week 1, participants completed the consent/pre-test visit and the targeting visit. The consent/pre-test visit entailed consent, screening questionnaires, and the pre-iTBS task sessions. Tasks included the no-shock, predictable-shock, unpredictable-shock (NPU) task (Fig. 1B), and the Sternberg working memory task (Fig. 1C). The targeting (MRI) visit, which was used to identify an individualized TMS target, included anatomical, resting and task (Sternberg) scans. During Weeks 2 and 4, subjects completed 2 days (2 sessions per day) of either active or sham iTBS, followed by a post-iTBS testing visit on the third day. The order of active and sham visits was counterbalanced across subjects. The post iTBS testing visit included the NPU and Sternberg WM tasks.

#### Consent visit procedure

First, subjects signed the informed consent form. They then completed the MRI safety form, the TMS adult safety screen (TASS), a medical history questionnaire, the coordinator administered the SCID, and an eligibility checklist. Subjects who met screening criteria continued to complete the demographics questionnaire, Montgomery-Asberg Depression Rating Scale (MADRS), the State/Trait Anxiety Inventory (STAI), and the Beck Anxiety Inventory (BAI). Afterwards, they completed the pre-stimulation test visit procedure.

#### Targeting visit procedure

Subjects were escorted to the scanner and given ear plugs, a button box, an emergency squeeze ball, and padding to minimize head movement. Next, structural scanning was completed from start to finish without intervention. Afterward, subjects then completed 1 run of the Sternberg WM task and 2 resting state runs.

#### Test visit procedure

Upon arrival, subjects were prepared for testing. Their skin was cleaned and prepped with an exfoliant gel. Afterward, electrodes were attached for electromyography (EMG), electrodermal activity (EDA), and shock delivery. Next subjects completed a shock workup and a startle habituation task. Subjects then completed 2 runs of the NPU task followed by 2 runs of the Sternberg+threat task.

#### TMS visit procedure

To prepare subjects for TMS, they were given a swimcap affixed with neuronavigation sensors, and scalp stimulation (sham) electrodes. The neuronavigation sensors were then registered to their MRI scan using fiducial points. On the first TMS visit, subjects’ motor threshold was obtained using the parameter estimation by sequential testing (PEST) procedure [31]. During each TMS session, subjects completed a single run of the Sternberg WM task followed by a 600-pulse train of iTBS and then another run of the Sternberg WM task. The purpose of including the Sternberg runs before and after the iTBS train was to activate and prime the working memory processes in the right dlPFC in an effort to control the subjects’ brain state during the stimulation [22]; however, it should be noted that the concept to use tasks to “control” brain state is still a speculative idea without clear testing. During each TMS visit, they were given 2 TMS sessions separated by a 30-minute break.

### Materials

#### NPU (Testing sessions)

During testing sessions, subjects did 2 runs of the NPU threat task, which consisted of alternating blocks of neutral (no shock), predictable (at risk for shock only during cue), and unpredictable threat (at risk for shock throughout) [10, 30, 32]. Predictable and Unpredictable blocks were separated by a neutral block to yield the following two block orders: **NPNUNUNP, NUNPNPNU**. Subjects were informed of the threat contingencies prior to the task, and the block type was displayed at the top of the screen. Blocks contained “cue” and intertrial interval (“ITI”) trials during which white noise was presented during the presence or absence of a visual cue, which were (8 s) simple colored (orange, teal, and purple) shapes (triangle, square, and pentagon). Colors and shapes varied across conditions. Each of the 4 Neutral blocks had 2 trials per conditions, while Predictable (x2) and Unpredictable (x2) blocks had 4 trials per condition for a total of 16 trials per condition across the two runs. Three shocks were presented during each run during either the cue (predictable condition) or the ITI (unpredictable condition) of a randomly selected trial. Subjects rated their anxiety from 0 (not anxious) to 10 (extremely anxious) throughout the task.

#### Sternberg + threat WM task (Testing sessions)

After the NPU runs, subjects completed 2 runs of the Sternberg + threat WM task, which consisted of a series of WM trials presented during safe (no shock) and threat (shock at any time) conditions, designed to test the effects of arousal on WM performance. Like the NPU task, subjects were informed of the contingencies prior to the task. Subjects were shown a blue circle during safe blocks and an orange circle during threat blocks. During these safe and threat blocks, subjects completed several “maintain” and “sort” WM trials. Each trial started with an instruction keyword to indicate the trial type. Then, subjects viewed a series of 5 letters, followed by a brief retention interval, where subjects were asked to either retain the letters in the order that they were presented (maintain) or rearrange the letters in alphabetical order (sort). After this interval, subjects were prompted with a letter/number combination, and they had to indicate with a button press whether the position of the letter in the series matched the number. Half of the trials were matches, and half were mismatches. The duration of the letter series (1.5–2.5 s), retention interval (6.5–8.5 s), and intertrial interval (5–8 s) were jittered across trials, while the duration of the instructions (1 s) and response prompt (3 s) were fixed. Two shock trials were presented in each run during random threat trials. Importantly, these “shock trials” were added to the design, and subsequently discarded from the analysis to avoid contamination by the shock delivery. Safe and threat blocks alternated and there were 2 of each block type per run. Block order was counterbalanced across runs. There were 3 trials per condition per block for a total of 12 trials per condition.

#### Sternberg WM task (targeting session)

During the targeting session, subjects completed a single run of the Sternberg WM task, while fMRI was recorded. There were no threat blocks and no shock during this targeting session. There were 12 trials each for the sort and maintain conditions. The duration of the letter series (1.5–2.5 s), retention interval (6.5–8.5 s), and intertrial interval (5–8 s) were jittered across trials, while the duration of the instructions (1 s) and response prompt (3 s) were fixed. All other aspects of the task were similar to the Sternberg+threat task.

#### Sternberg WM task (TMS sessions)

During the TMS sessions, subjects completed short runs of the Sternberg WM task before and after iTBS administrations. Like in the targeting session, there was no shock and no threat. There were 4 trials each for the sort and maintain conditions pre run. All other aspects of the task were similar to the targeting session run.

#### White noise

During the NPU task, subjects received occasional 40-ms, white noise presentations (103-dB, instantaneous rise time), via standard over-the-ear headphones (Sennheiser HD280PRO, Sennheiser electronic GmbH & Co., Wedemark, Germany) [33].

#### Startle habituation (Testing sessions)

At the start of each testing session, subjects received 9 unsignaled white noise presentations spaced approximately ~17 s apart.

#### Electromyography

Facial EMG was recorded from 15 × 20 mm hydrogel coated vinyl electrodes (Rhythmlink #DECUS10026; Columbia, SC) attached just below the left eye (orbicularis oculi muscle) and sampled at 2000 Hz using a Biopac MP160 unit (Biopac; Goleta, CA) via.

#### EMG processing

EMG data were processed using the analyze_startle package developed by Dr. Balderston (https://github.com/balders2/analyze_startle). The EMG signal was bandpass filtered from 30 to 300 Hz, rectified, and smoothed using a 20-ms sliding window. Startle responses were extracted scored as the peak (max during the 20 ms to 120 ms post-noise burst window) – the baseline (50 ms pre-noise window). Raw startle responses were then converted to t-scores (tx = [Zx × 10]  +  50). Trials with excess noise (baseline SD > 2× run SD) were counted as missing data. Trials with no blink (peak < baseline range) trials were coded as 0.

#### Shock

Shocks were delivered to the subject’s left wrist via disposable 11 mm Ag/AgCl electrodes (Biopac Item number EL508; Goleta, CA), spaced ~2 cm apart. The shock stimulus consisted of a 100 ms train of 2 ms pulses delivered at 200 Hz using a using a DS7A constant current stimulator (Digitimer #DS7A, Ft. Lauderdale, FL). The intensity of the shock was calibrated prior to each testing session using an individualized workup procedure. Subjects rated each shock on a scale from 1 (not uncomfortable) to 10 (uncomfortable but tolerable) until the subject reached their reported “level 10”. Subsequent shocks were delivered at the same level for the remainder of the testing session.

#### Scans

MRI data was acquired using a 3 Tesla Siemens Prisma scanner with a 64-channel head coil (Erlangen, Germany). Structural scans included a T1-weighted MPRAGE (TR  =  2200 ms; TE  =  4.67 ms; flip angle = 8°) with 160, 1 mm axial slices (matrix = 256 × 256; field of view (FOV)  =  240 mm × 240 mm), and a T2-weighted image (TR  =  3200 ms; TE  =  563 ms; flip angle = variable) with 160, 1 mm sagittal slices (matrix = 256 mm × 256 mm; FOV  =  240 mm × 240 mm). Each task and rest run included 615 whole-brain BOLD images (TR  =  800 ms; TE  =  37 ms; flip angle = 52°; Multi-band acceleration factor = 8) comprised of 72, 2 mm axial slices (matrix = 104 × 104; FOV  =  208 mm × 2008 mm) aligned to the AC-PC line.

#### fMRI Pre-processing

Sternberg WM data were processed using the afn_proc.py script distributed with the AFNI software package [34]. The following the following preprocessing blocks: tshift, align, volreg, blur, mask, scale, regress were used. During preprocessing, 1) the images were slice time corrected, 2) images were aligned to the T1 data using an Local Pearson Correlation cost function, 3) individual volumes were registered to the image with the fewest outliers, 4) images were blurred with a 2 mm Gaussian kernel, 5) images were then masked using the union of the EPI brain mask and the skull-stripped T1, 6) they were then scaled so the mean of each voxel timeseries was 100. The first 4 TRs and TRs with greater than 0.5 mm displacement or greater than 15% of voxels registered as outliers were scrubbed from the timeseries prior to the GLM. The first-level GLM included regressors of no interest corresponding to the 6 primary motion vectors and their derivatives, and a set of polynomial regressors to model the baseline. Task events were modeled with variable duration blocks to account for jittering in the timing of the events.

#### Head modeling

The SimNIBS software package (Version 2.1) was used to create finite element models representing the head and coil geometries from the T1 and T2 scans [35]. Images were first segmented into tissue compartments (i.e. scalp, skull, CSF, gray matter, and white matter), then meshed using gmsh subroutines [36].

#### Target localization

**Fig. 2:**
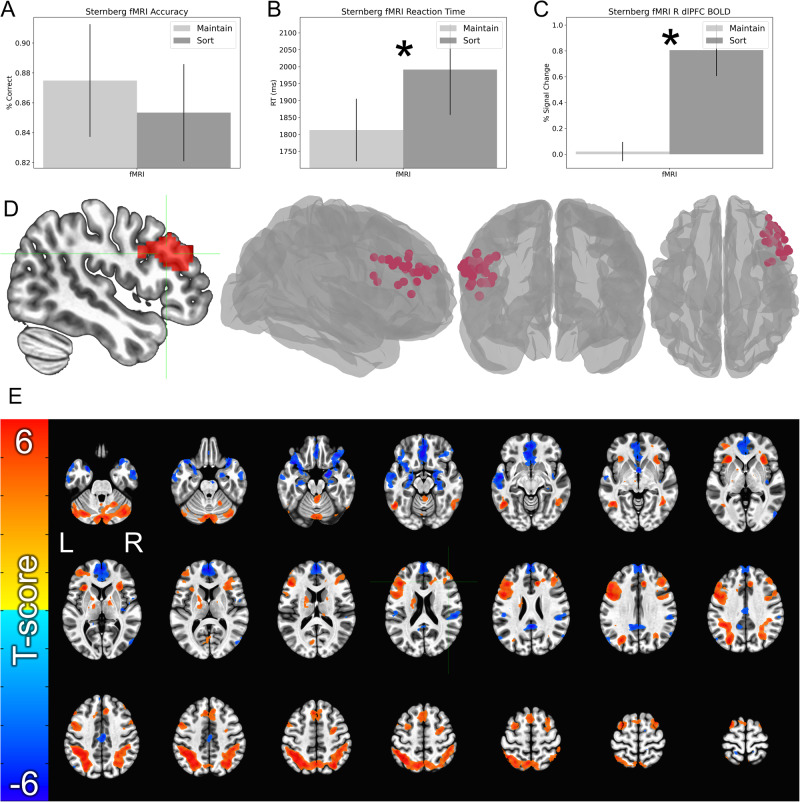
dlPFC BOLD and performance from the Sternberg targeting run. **A** Accuracy during the sort and maintain trials. **B** Reaction time during the sort and maintain trials. **C** BOLD data extracted from the group-level mask shown in panel **D**. **D** Solid mask represents the group level region of interest that we used to confine the single subject fMRI peaks. Spheres represent the single-subject peaks for WM-related activity during the Sternberg WM task. **E** Whole-brain BOLD responses for the Sort > Maintain contrast. Cool colors represent maintain > sort effects. Warm colors represent sort > maintain effects. Bars represent the mean ± SEM. Asterisks indicate that *p* < 0.05.

Data from the Sternberg WM task was used to identify the target coordinates for each subject (Fig. 2D) [29, 30]. BOLD maps from the retention interval were masked with a functional region of interest encompassing the right dlPFC, defined from a group-level analysis using a previously collected Sternberg WM dataset [3, 37]. Then activity was contrasted across sort and maintain trials and the peak voxel within this mask was extracted and used as a target. Target site coordinates were then projected to the scalp mesh using a nearest neighbor search.

#### Electric-field (e-field) calculations

For the e-field models, the roll and pitch of the coil were defined perpendicular to the cortex at the site of stimulation. There were multiple (24) e-field models conducted at evenly spaced (15 degrees) yaw orientations centered on the scalp target. The e-field magnitude was then averaged within the right dlPFC region of interest, and the yaw orientation corresponding to the maximal E-field within this region of interest was used for stimulation [30, 37].

#### Active stimulation

A Magventure MagPro X100 stimulator with a B65AP (active/placebo) figure-8 coil was used for the iTBS sessions. We masked the active and sham sides of the coil and assigned them blinded labels (e.g. A = active, B = sham). The label key was maintained by an employee of the center that was not a member of the study staff and not directly involved in the collection or analysis of the data. All study staff were blinded to the label assignments.

#### Sham stimulation

The sham side of the B65AP has the same visual characteristics as the active side. However, it has an internal magnetic shield that limits the output (sham output <5% of the active output). During sham sessions, electric stimulation was delivered to the scalp concurrent with each TMS pulse to match the sensation of the active stimulation. Importantly, the sham e-stim pulse was titrated to match the sensation of the active TMS pulse. To deliver the e-stim, 15 × 20 mm hydrogel coated vinyl electrodes (Rhythmlink #DECUS10026; Columbia, SC) were attached to the scalp, adjacent to the stimulation site. Active and sham e-stim cables were created to deliver (or not) e-stim pulses to the scalp only during the sham TMS sessions. These cables were blinded and assigned labels to match the corresponding coil side so that each session always included either active TMS or active e-stim, but never both.

#### Motor threshold determination

Motor threshold was determined from EMG recordings of the first dorsal interosseous muscle using the adaptive PEST algorithm [38]. Because the motor threshold procedure required active stimulation, a B65 coil was used. Importantly, this coil was calibrated against the B65AP coil to ensure comparable output during the iTBS sessions.

#### iTBS parameters

During each iTBS session, ten 60-pulse iTBS trains were delivered at 100% of RMT. The trains consisted of 3 50 Hz bursts repeated at intervals of 200 ms (5 Hz) for 2 s of stimulation separated by 8 s gaps, for a total of 600 pulses per session.

#### Neuronavigation

We used the Brainsight (Rogue Research Inc, Montreal, Canada) frameless stereotaxic neuronavigation system for neuronavigation. We generated nifti files aligned to the subject’s native space T1 image to mark the target and orientation of the coil. These files were loaded into Brainsight, and scalp and cortical surfaces were generated from the T1. Prior to TMS, the subject’s head was co-registered to the T1 using fiducial points at the nasion and tragi, and 50–100 refinement points distributed evenly across the scalp. The subject’s target and orientation files were used to create a Brainsight trajectory, the TMS was delivered according to this trajectory, and the accuracy was recorded by the Brainsight software.

### Statistical analyses

#### Targeting session performance

Percent correct and reaction time were averaged across trials for each subject and condition. To calculate the dlPFC BOLD effect, first-level GLM betas from the retention interval were extracted using voxels from the dlPFC targeting mask. Paired sample (Sort > Maintain) t-tests then conducted on these values.

#### Targeting session whole-brain BOLD

As a manipulation check, we conducted voxelwise analyses of BOLD responses during the retention interval at the whole-brain level. We extracted first-level GLM beta values and performed a paired-sample t-test using the AFNI program 3dttest++. We used the cluster-based thresholding, as implemented by the AFNI program 3dClustSim [39] with a t-tailed voxelwise p-value of 0.001, a non-Gaussian (i.e. autocorrelation function) [40] estimation of the smoothness of the BOLD data, and clusters comprised of voxels with adjoining faces or edges. We ran 10,000 Monte Carlo simulations with these parameters, which resulted in a minimum cluster size of 33, 2-mm isotropic voxels.

#### Testing session Sternberg threat WM performance

Percent correct and reaction time were averaged across trials for the sort and maintain trials during safe and threat blocks. WM-related effects were examined by creating WM-related difference scores (Sort – Maintain). A 2 (Coil: Active vs. Sham) ×2 (Condition: Safe vs. Threat) repeated measures ANOVA was conducted on these difference scores.

#### NPU anxiety ratings and startle

Anxiety ratings at the onset of each WM presentation were extracted and averaged across trials. EMG data were processed, and startle magnitude was averaged across trials. For both ratings and startle, APS (anxiety-potentiated startle) and FPS (fear-potentiated startle), difference scores were created from the rating and startle data (FPS: Predictable Cue – Predictable intertrial interval; APS_ITI: Unpredictable intertrial interval – Neutral intertrial interval). A 2 (Coil: Active vs. Sham) ×2 (Trial type: FPS vs. APS_ITI) repeated measures ANOVA was conducted on these values.

Outliers (i.e. values greater than 2 SD) were truncated to 2 standard deviations from the mean (i.e. *x*(|*x* > M ± 2*SD|) = M ± 2*SD) for all measures. Significant 2-way interactions and multi-level 1-way main effects were probed using *post hoc p*aired-sample t-tests.

## Results

### Sternberg performance during fMRI targeting session

As a manipulation check, we measured accuracy, reaction time, and BOLD during the pre-TMS fMRI targeting session. We sampled the BOLD activity using the single subject targets identified by the targeting algorithm. For all 3 measures, we used a paired-sample t-test to compare maintain and sort trials (Fig. 2).

#### Accuracy and reaction time

We found no significant difference for accuracy (t(24) = 0.78; *p* = 0.443; *d* = 0.16; Fig. 2A), but a significant main difference for reaction time (t(24) = 2.13; *p* = 0.044; *d* = 0.43; Fig. 2B), such that subjects were faster on the maintain trials than on the sort trials, as shown in our previous studies [3, 5, 29].

#### dlPFC BOLD

**Fig. 3:**
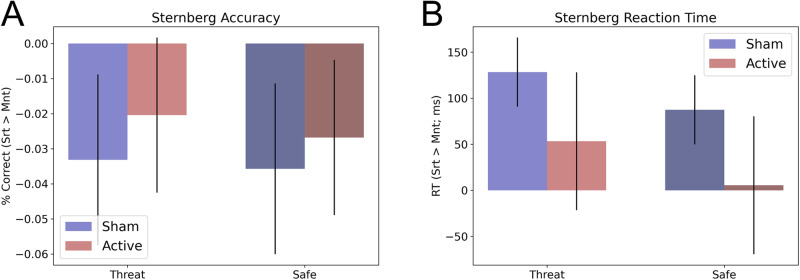
Accuracy and reaction time during the Sternberg WM task. **A** Percent correct during the Sternberg+threat WM task. **B** Reaction time during the Sternberg + threat WM task. Bars represent the mean ± SEM.

As designed, we found significantly greater BOLD activity at the target site for the sort compared to the maintain trials (t(27) = 4.41; *p* < 0.001; *d* = 0.83; Fig. 2C), indicating that our fMRI-guided targeting approach was successful at identifying single subject targets for working memory manipulation. See Fig. 3 for a distribution of these targets in MNI space.

### Whole-brain BOLD during fMRI targeting session

In addition to sampling BOLD at the single subject target sites, we also examined the effect of working memory manipulation on voxel-wise whole-brain BOLD activity during the retention interval. As expected, these results generally replicated previous work showing broad activation in frontal and parietal regions thought to be important for attention and working memory, as well as deactivation of regions within the default mode network (e.g. ventromedial prefrontal cortex, posterior cingulate cortex, hippocampus, etc.; Fig. 2E and Table 1) [41–45].

**Table 1:** Coordinates from sort vs. maintain contrast.

Region label	Coordinates			t-score	Volume
	X	Y	Z		
Left Superior Parietal Lobule	−33	−59	47	9.27	47583
Right Cerebellum VIII	4	−70	−40	7.71	27450
Left Rectal Gyrus	−2	36	−16	−6.32	23325
Left Inferior Frontal Gyrus p. Triangularis	−41	31	22	6.98	20534
Right ParaHippocampal Gyrus	18	−4	−19	−6.54	11482
Right Middle Cingulate Cortex	11	30	27	6.50	9279
Right Insula Lobe	35	18	−3	6.21	8594
Left Cerebellum Crus 2	−32	−74	−31	6.94	7900
Left Temporal Pole	−42	19	−22	−7.85	7027
Left Middle Temporal Gyrus	−53	−7	−10	−6.06	6149
Right Superior Frontal Gyrus	33	10	65	5.69	3651
Left Pallidum	−24	−15	−3	5.75	2841
Left Inferior Temporal Gyrus	−52	−58	−14	6.82	2725
Right Posterior Cingulate Cortex	3	−46	25	−5.83	2707
Left Superior Frontal Gyrus	−27	7	69	6.45	2585
Right Superior Temporal Gyrus	56	−28	20	−5.35	2526
Left Insula Lobe	−29	23	0	7.62	2394
Right Inferior Temporal Gyrus	43	−53	−4	6.26	2318
Right Precentral Gyrus	38	3	32	6.06	2088
Right Middle Cingulate Cortex	4	−16	39	−5.41	1885
Cerebellar Vermis 3	1	−48	−19	5.56	1165
Right Middle Occipital Gyrus	53	−77	3	−6.36	789
Left Calcarine Gyrus	−7	−76	14	4.43	626
Left SupraMarginal Gyrus	−49	−26	24	−5.04	614
Left Angular Gyrus	−55	−70	30	−4.19	600
Right Insula Lobe	37	−16	4	−5.17	408
Right Thalamus	14	−3	5	5.66	397
Left Superior Parietal Lobule	−17	−43	76	−4.85	341
Right Middle Temporal Gyrus	58	−65	24	−4.40	261
Right Rolandic Operculum	56	3	7	−4.28	255
Right Caudate Nucleus	20	2	16	4.69	189
Right Insula Lobe	40	−8	−10	−4.33	171
Right Thalamus	13	−28	18	7.01	137
Cerebellar Vermis 9	−2	−57	−31	4.61	120
Right Anterior Cingulate Cortex	25	37	0	4.05	111
Right Middle Temporal Gyrus	65	−9	−19	−4.10	104
Brainstem	0	−37	−41	4.31	84
Right Superior Frontal Gyrus	24	49	−4	4.09	72
Brainstem	7	−17	−25	−5.04	72
Left Cerebellum VIII	−28	−37	−44	5.01	71
Left Precuneus	−15	−43	6	−4.36	52
Right Postcentral Gyrus	15	−40	66	−3.91	44
Right SMA	13	−5	67	−4.26	44
Left Thalamus	−7	−14	−4	4.14	39
Left Cerebellum VIII	−15	−64	−42	4.14	38
Right Inferior Frontal Gyrus p. Opercularis	34	17	31	3.98	37
Left Cerebellum VIII	−28	−58	−34	3.91	33
Left Insula Lobe	−37	−15	24	−4.04	33

### Effect of iTBS to the right dlPFC on Sternberg performance

Subjects performed the Sternberg task 24 h after the last session of (active/sham) iTBS. The task included periods of safety and threat. Accordingly, we used a 2 (coil: active vs. sham) ×2 (Condition: safe vs. threat) repeated measures ANOVA.

#### Accuracy

For accuracy we found no significant main effect of either coil (f(1,27) = 0.29; *p* = 0.594; eta-squared = 0.01; Fig. 3A) or condition (f(1,27) = 0.07; *p* = 0.792; eta-squared = 0), and no coil by condition interaction (f(1,27) = 0.01; *p* = 0.943; eta-squared = 0).

#### Reaction time

We observed a similar pattern for reaction time, with no main effects (coil: f(1,27) = 1.36; *p* = 0.253; eta-squared = 0.05; condition: f(1,27) = 1.1; *p* = 0.304; eta-squared = 0.04; Fig. 3B) or interactions (f(1,27) = 0.01; *p* = 0.946; eta-squared = 0).

### Effect of iTBS to the right dlPFC on anxiety during NPU task

Subjects also completed the NPU task 24 h after the last session of (active/sham) iTBS. We recorded both anxiety ratings and startle responses during the task. We examined the effect of iTBS on the standard FPS and APS responses generated from this task using a 2 (coil: active vs. sham) ×2 (response: FPS vs. APS) repeated measures ANOVA.

#### Ratings

**Fig. 4:**
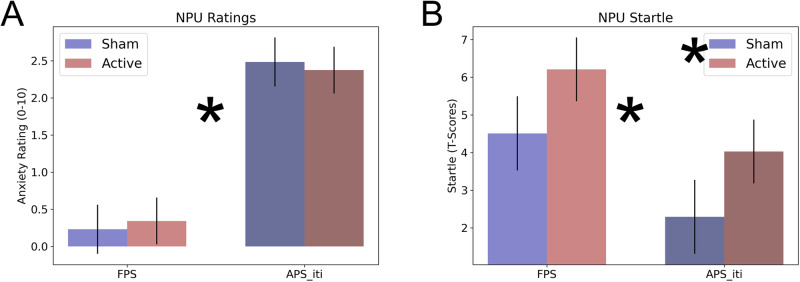
Anxiety ratings and startle during the NPU threat task. **A** Anxiety ratings reported on a scale from 0 to 10. **B** Potentiated startle represented as T-scores. For both measures, difference scores were calculated to correspond to Fear (FPS: Predictable Cue – Predictable ITI), Anxiety during the ITI (APS_iti: Unpredictable ITI – Neutral ITI). Bars represent the mean ± SEM. Asterisks indicate that *p* < 0.05.

For the ratings, we found no significant main effect of coil (f(1,27) = 0; *p* = 0.988; eta-squared = 0; Fig. 4A) and no coil by response type interaction (f(1,27) = 1.65; *p* = 0.21; eta-squared = 0.06). These results suggested that the coil did not influence subjective anxiety levels, consistent with the double-blind design of the study. However, we did find a significant main effect of response type (f(1,27) = 46.52; *p* < 0.001; eta-squared = 0.63), suggesting that subjects increased their ratings more from Neutral to Unpredictable than they did from the Predictable ITI to the Predictable cue periods.

#### Startle

For startle, we found a significant main effect for both coil (f(1,27) = 5.1; *p* = 0.032; eta-squared = 0.16; Fig. 4B) and response type (f(1,27) = 10.48; *p* = 0.003; eta-squared = 0.28). However, we found no coil by response type interaction (f(1,27) = 0; *p* = 0.979; eta-squared = 0). Consistent with previous studies, FPS responses were greater than APS responses [10, 30]. More importantly, both FPS and APS increased after active compared to sham iTBS.

## Discussion

In the current study, we administered 4 sessions of active or sham iTBS to the right dlPFC of healthy volunteers and measured the effect of this stimulation on fear and anxiety potentiated startle 24-h post stimulation. We targeted the right dlPFC using fMRI activity during the maintenance interval of the Sternberg WM paradigm [3–6] and used e-field modeling to optimize and individualize the coil orientation [37]. We used a strong within-subject design with an appropriate sample size and a large washout period to avoid carry-over effects. We used the well-validated NPU threat paradigm to elevate arousal during the pre and post stimulation testing periods and included both objective (startle) and subjective (ratings) measures of anxiety as primary outcome measures [46–53]. Contrary to our hypothesis, we found that active stimulation compared to sham iTBS increased, rather than decreased, the magnitude of the anxiety potentiated startle.

This study is the latest in a series of studies conducted by our group dating back nearly a decade, aimed at understanding the relationships between anxiety, cognition, and the right dlPFC. We initially observed a link between anxiety and right dlPFC activity in a study examining working memory performance in anxiety patients using the N-back task [12]. We found that anxiety patients generally had reduced right dlPFC activity in this task, suggesting a potential deficit in right dlPFC processing [3]. Consistent with this anxiety/cognition link, we found that threat of shock increased right dlPFC activation on a pattern separation task, but only during lure trials that required a difficult mental comparison with the target stored in WM [11]. Interestingly, this right dlPFC activity also correlated with performance in this study [11]. In another study, we found that simple visual cues (e.g. geometric shapes) could elicit dlPFC activity when presented during unpredictable threat blocks [10]. Critically, the dlPFC activity in that study was also negatively correlated with startle levels outside the scanner [10]. Consistent with the idea that the right dlPFC can help regulate anxiety, further work from our group showed that tasks that drive dlPFC activity (i.e. complex WM tasks) could also reduce anxiety potentiated startle [5]. Finally, our results are consistent with a broader set of findings suggesting that emotion regulation activates the dlPFC [7–9], dlPFC stimulation can facilitate emotion regulation [54], and that lesions of the dlPFC can impair emotion regulation [55].

Our general approach is to use these psychophysiology and neuroimaging studies to generate hypotheses about brain/behavior connections, and to then test these hypotheses using causal neuromodulation techniques. Based on the top-down inhibition model, we initially hypothesized that boosting right dlPFC activity would lead to greater inhibition and thus reduce anxiety potentiated startle [56–58]. We tested this by recording APS before and after 10 Hz stimulation in a within-session paradigm [30]. Consistent with our current results, but counter to our initial hypothesis, we found increases in APS following active but not sham 10 Hz stimulation [30]. Since these results were surprising, we wanted to rule out any potential off-target within-session effects by testing at 24 h. Accordingly, for our next study we recorded APS before and 24 h after 4 sessions of cTBS to the right dlPFC [29]. Given that previous research in anxious patients showed efficacy for right lateralized low-frequency stimulation [59–64], and that cTBS is similarly thought to induce long term depression like effects [23–28], we expected to show reduced APS following active but not sham stimulation. Again, counter to our initial hypothesis, we found increased APS 24 h after active but not sham cTBS. These results were potentially consistent with the top-down inhibition model, in that they showed that potential long term depression like effects in right dlPFC also led to potential decreases in top-down inhibition and net increases in anxiety potentiated startle [56–58]. However, they potentially conflicted with our earlier findings and still offered no direct path toward TMS-induced startle reduction. This brings us to our current work. Again, based on the top-down inhibition model and our previous cTBS results, we hypothesized that if inducing long term depression like effects in the right dlPFC results in a net increase in startle then inducing long term potentiation like effects in the right dlPFC should result in a net decrease in startle (see caveats related to these assumptions below). Accordingly, we recorded APS before and 24 h after active or sham iTBS. We expected to show decreases in APS following active but not sham stimulation. Once again, counter to our initial hypothesis for this study, but consistent with all previous results, we observed the opposite. That is, APS was increased following active but not sham iTBS. It is critical to note that targeting, dosing, motor thresholding, and neuronavigation methods were all held constant across these studies.

Although the findings are similar across the cTBS and iTBS (current) studies, we believe it is premature to imply that cTBS and iTBS have equivalent effects on anxiety potentiated startle, as there may be subtle differences to which we are not sensitive. Additionally, while it might be tempting to conclude from these studies that any intervention that affects right dlPFC activity will increase anxiety, we believe that this conclusion is premature. The issue of healthy controls vs. patients is also a very important topic. For instance, there are several trials in patients with anxiety disorders suggesting that 1 Hz rTMS to the right dlPFC can reduce anxiety symptoms [59–64]. In a small trial delivering bilateral-sequential rTMS to patients with MDD and comorbid GAD, 11 out of 13 achieved remission for their anxiety symptoms [61]. Similarly, retrospective studies of anxious MDD patients showed that depression protocols, including 1 Hz stimulation to the right dlPFC could reduce anxiety symptoms [62–64]. Finally, anxiety patients receiving 1 Hz rTMS have shown greater improvements in response and remission rates compared to sham stimulation [59, 60]. In PTSD, there’s some evidence that high frequency stimulation to either the left or right dlPFC is therapeutic [65, 66]. Similarly, iTBS to the right dlPFC can reduce anger [67] and improve PTSD symptoms [68, 69]. Based on these results, one might conclude that healthy control studies might not provide the optimal guidance for TMS studies in patients. As discussed below, TMS may differentially impact arousal levels in healthy controls and patients by differentially impacting working memory related processing in the dlPFC. Additionally, the healthy volunteer studies described here (including the current study) all use startle as an outcome measure. Importantly, startle is a physiological index of arousal that, at best, only reflects a single symptom dimension experienced by patients with anxiety, and may not capture the most critical subjective features of anxiety [70]. This may mean that clinically it may be more important to target the subjective features of anxiety. There is some support for this conclusion coming from studies showing that high frequency left dlPFC stimulation can improve anxiety symptoms in anxious and depressed individuals [71].

Our results also raise questions regarding the behavioral significance of patterned theta burst stimulation. That is, we find little difference in behavioral outcomes when we stimulate the right dlPFC with continuous vs. intermittent TBS. Given that we cannot directly measure changes in synaptic plasticity in such non-invasive studies, we cannot examine the differential effects of continuous vs. intermittent TBS on synaptic changes. Instead, we raise the testable hypothesis that for dlPFC stimulation, perhaps the specificity of the changes matter more than the direction. According to this hypothesis, it may be possible to modulate the behavioral effects of TBS by modifying the context during which the TBS is delivered. That is, it may be possible to use contextual elements to activate specific synaptic connections so that they are more plastic when the TBS is administered [22].

Although this specificity hypothesis may account for why cTBS and iTBS induce similar effects, it is not sufficient to explain why these effects are opposite of what we expected (i.e. increased startle). One possible explanation for these counterintuitive results is that modulating right dlPFC activity interferes with ongoing emotion regulation. Indeed, there are strong data supporting the idea that both the left and right dlPFC play a role in emotion regulation [7–9]. However, the observation that low-frequency (inhibitory) rTMS to the right dlPFC tends to reduce anxiety symptoms in depressed individuals runs counter to the emotion regulation hypothesis [59–64]. Instead, we speculate that the primary functional domain of the right dlPFC may be non-verbal emotional working memory, and that the behavioral effects of neuromodulation to the right dlPFC may depend on the valence of the emotional content in these emotional working memory stores, which might differ in healthy individuals compared to patients with anxiety [72]. Support for this comes from work showing Left lateralized activity for verbal WM, right lateralized activity for spatial WM [73]. Although speculative, this model raises the testable hypothesis that there may be differential effects of rTMS in patients versus healthy controls because rTMS may interfere with the maintenance of negative emotional content in patients and positive emotional content in healthy subjects. In any case, future work should explicitly explore laterality effects by directly comparing the effects of left and right dlPFC stimulation on anxiety expression. Additionally, given the untested and speculative nature of using tasks to control brain state during stimulation, future studies should include both verbal and non-verbal measures of mood and anxiety and determine whether these facilitate stimulation.

## Strengths and weaknesses

Among the strengths of our study, we used a robust within-subject crossover design that included an adequate sample size and a 1-week washout period to test the effects of active vs. sham stimulation. We used an individualized fMRI-guided targeting approach based on the Sternberg WM paradigm, which is known to strongly drive BOLD activity in the dlPFC [3–6]. We used e-field modeling to optimize and individualize the orientation of the TMS coil in order to maximize the e-field magnitude at the site of stimulation [37]. We used a well-validated, extensively researched unpredictable threat of shock paradigm, which is known to robustly induce sustained levels of elevated arousal [46–53]. We then measured this anxiety using reliable objective (startle) and subjective (ratings) measures that allowed us to distinguish between mechanistic changes in arousal (i.e. differences in APS) as a function of cTBS and off-target expectancy effects (i.e. potential changes in anxiety ratings) due to placebo responding [29, 30]. Finally, we used a double-blind design with an active scalp-stimulating sham condition to minimize unintentional unblinding [74–77].

Despite these strengths, it should be noted that this study was a mechanistic trial conducted in healthy volunteers. Accordingly, it would be premature to generalize these findings to the symptoms and experiences of individuals suffering from anxiety disorders. Future work should be conducted in these individuals to understand the degree to which the mechanisms of anxiety expression and regulation differ in clinical anxiety. Finally, given the clinical success of 1 Hz stimulation, in anxious individuals [59–64], future research should be conducted using this pattern of stimulation in non-depressed anxiety patients. It should also be noted that TBS protocols are associated with considerable interindividual variability in clinical and physiological measures [78]. Although we tried to mitigate this variability by using individualized fMRI and e-field based targeting and online neuronavigation, future studies should replicate and extend these findings to larger samples with additional convergent outcome measures.

## Conclusions

Here we administered active vs. sham iTBS to the right dlPFC and measured changes in anxiety potentiated startle. We found that active but not sham iTBS led to an increase rather than decrease in startle. These results extend our previous work and highlight the mechanistic link between right dlPFC functioning and arousal, and further suggest that the specificity (i.e. cells or connections targeted) of intervention may be more important than the pattern of intervention, raising the testable hypothesis that context dependent stimulation may improve rTMS efficacy. Future research should extend these findings to anxiety patients.

## Supplementary information

Consort Diagram

## References

[1] Kessler RC, Chiu WT. Prevalence, severity, and comorbidity of 12-month DSM-IV disorders in the National Comorbidity Survey Replication. Arch Gen. 2005;62:617–27. 10.1001/archpsyc.62.6.617 PMC2847357 15939839

[2] Eysenck MW, Derakshan N, Santos R, Calvo MG. Anxiety and cognitive performance: attentional control theory. Emot Wash DC. 2007;7:336–53. 10.1037/1528-3542.7.2.336 17516812

[3] Balderston NL, Flook E, Hsiung A, Liu J, Thongarong A, Stahl S, et al. Anxiety Patients Rely On Bilateral DLPFC Activation During Verbal Working Memory. Soc Cogn Affect Neurosci. 2020:1–11. 10.1093/scan/nsaa146 PMC7759210 33150947

[4] Balderston NL, Hsiung A, Liu J, Ernst M, Grillon C. Reducing state anxiety using working memory maintenance. J Vis Exp. 2017. 10.3791/55727. 10.3791/55727 PMC5612581 28745646

[5] Balderston NL, Quispe-Escudero D, Hale E, Davis A, O’Connell K, Ernst M, et al. Working memory maintenance is sufficient to reduce state anxiety. Psychophysiology. 2016;53:1660–8. 10.1111/psyp.12726 PMC5061597 27434207

[6] Sternberg S. High-speed scanning in human memory. Science. 1966;153:652–4. 5939936 10.1126/science.153.3736.652

[7] Gray JR, Braver TS, Raichle ME. Integration of emotion and cognition in the lateral prefrontal cortex. Proc Natl Acad Sci USA. 2002;99:4115–20. 11904454 10.1073/pnas.062381899 PMC122657

[8] Liberzon I, Taylor SF, Fig LM, Decker LR, Koeppe RA, Minoshima S. Limbic activation and psychophysiologic responses to aversive visual stimuli. Interaction with cognitive task. Neuropsychopharmacology. 2000;23:508–16. 11027916 10.1016/S0893-133X(00)00157-3

[9] Beauregard M, Lévesque J, Bourgouin P. Neural Correlates of Conscious Self-Regulation of Emotion. J Neurosci 2001;21:RC165 LP–RC165. 11549754 10.1523/JNEUROSCI.21-18-j0001.2001 PMC6763007

[10] Balderston NL, Liu J, Roberson-Nay R, Ernst M, Grillon C. The relationship between dlPFC activity during unpredictable threat and CO2-induced panic symptoms. Transl Psychiatry. 2017;7:1266. 29213110 10.1038/s41398-017-0006-5 PMC5802456

[11] Balderston NL, Hsiung A, Ernst M, Grillon C. Effect of Threat on Right dlPFC Activity during Behavioral Pattern Separation. J Neurosci. 2017;37:9160–71. 28842415 10.1523/JNEUROSCI.0717-17.2017 PMC5607464

[12] Balderston NL, Vytal KE, O’Connell K, Torrisi S, Letkiewicz A, Ernst M, et al. Anxiety Patients Show Reduced Working Memory Related Dlpfc Activation During Safety and Threat. Depress Anxiety. 2016;12:1–12. 10.1002/da.22518 PMC5079837 27110997

[13] White LK, Sequeira S, Britton JC, Brotman MA, Gold AL, Berman E, et al. Complementary Features of Attention Bias Modification Therapy and Cognitive-Behavioral Therapy in Pediatric Anxiety Disorders. Am J Psychiatry. 2017;174:775–84. 28407726 10.1176/appi.ajp.2017.16070847 PMC6343478

[14] Vytal KE, Arkin NE, Overstreet C, Lieberman L, Grillon C. Induced-anxiety differentially disrupts working memory in generalized anxiety disorder. BMC Psychiatry. 2016;16:62. 26976146 10.1186/s12888-016-0748-2 PMC4791753

[15] Rapee RM. Generalized anxiety disorder: A review of clinical features and theoretical concepts. Clin Psychol Rev. 1991;11:419–40.

[16] Price RB, Eldreth DA, Mohlman J. Deficient prefrontal attentional control in late-life generalized anxiety disorder: an fMRI investigation. Transl Psychiatry. 2011;1:e46. 22833192 10.1038/tp.2011.46 PMC3309492

[17] Ansari TL, Derakshan N, Richards A. Effects of anxiety on task switching: Evidence from the mixed antisaccade task. Cogn Affect Behav Neurosci. 2008;8:229–38. 18814460 10.3758/cabn.8.3.229

[18] White LK, McDermott JM, Degnan KA, Henderson HA, Fox NA. Behavioral Inhibition and Anxiety: The Moderating Roles of Inhibitory Control and Attention Shifting. J Abnorm Child Psychol. 2011;39:735–47. 21301953 10.1007/s10802-011-9490-x PMC3624966

[19] Derakshan N, Ansari TL, Hansard M, Shoker L, Eysenck MW. Anxiety, Inhibition, Efficiency, and Effectiveness. Exp Psychol. 2009;56:48–55. 19261578 10.1027/1618-3169.56.1.48

[20] Shi R, Sharpe L, Abbott M. A meta-analysis of the relationship between anxiety and attentional control. Clin Psychol Rev. 2019;72:101754. 31306935 10.1016/j.cpr.2019.101754

[21] Berggren N. Anxiety and apprehension in visual working memory performance: no change to capacity, but poorer distractor filtering. Anxiety Stress Coping. 2020;33:299–310. 32126798 10.1080/10615806.2020.1736899

[22] Deng Z-D, Luber BM, Balderston NL, Velez Afanador M, Noh MMM, Thomas J, et al. Device-Based Modulation of Neurocircuits as a Therapeutic for Psychiatric Disorders. Annu Rev Pharmacol Toxicol. 2020;60:591–614. 31914895 10.1146/annurev-pharmtox-010919-023253 PMC8100981

[23] Barr DS, Lambert NA, Hoyt KL, Moore SD, Wilson WA. Induction and reversal of long-term potentiation by low- and high- intensity theta pattern stimulation. J Neurosci. 1995;15:5402–10. 7623162 10.1523/JNEUROSCI.15-07-05402.1995 PMC6577862

[24] Suppa A, Huang YZ, Funke K, Ridding MC, Cheeran B, Di Lazzaro V, et al. Ten Years of Theta Burst Stimulation in Humans: Established Knowledge, Unknowns and Prospects. Brain Stimulat. 2016;9:323–35. 10.1016/j.brs.2016.01.006 26947241

[25] Neves G, Cooke SF, Bliss TVP. Synaptic plasticity, memory and the hippocampus: a neural network approach to causality. Nat Rev Neurosci. 2008;9:65–75. 18094707 10.1038/nrn2303

[26] Dudek SM, Bear MF. Homosynaptic long-term depression in area CA1 of hippocampus and effects of N-methyl-D-aspartate receptor blockade. Proc Natl Acad Sci USA. 1992;89:4363–7. 1350090 10.1073/pnas.89.10.4363 PMC49082

[27] Dudek SM, Bear MF. Bidirectional long-term modification of synaptic effectiveness in the adult and immature hippocampus. J Neurosci. 1993;13:2910–8. 8331379 10.1523/JNEUROSCI.13-07-02910.1993 PMC6576673

[28] Huang YZ, Rothwell JC, Chen RS, Lu CS, Chuang WL. The theoretical model of theta burst form of repetitive transcranial magnetic stimulation. Clin Neurophysiol. 2011;122:1011–8. 20869307 10.1016/j.clinph.2010.08.016 PMC3046904

[29] Teferi M, Makhoul W, Deng ZD, Oathes DJ, Sheline Y, Balderston NL. Continuous Theta-Burst Stimulation to the Right Dorsolateral Prefrontal Cortex May Increase Potentiated Startle in Healthy Individuals. Biol Psychiatry Glob Open Sci. 2023;3:470–9. 37519467 10.1016/j.bpsgos.2022.04.001 PMC10382694

[30] Balderston NL, Beydler EMEM, Roberts C, Deng Z-D, Radman T, Lago T, et al. Mechanistic link between right prefrontal cortical activity and anxious arousal revealed using transcranial magnetic stimulation in healthy subjects. Neuropsychopharmacology. 2020;45:694–702. 31791039 10.1038/s41386-019-0583-5 PMC7021903

[31] Mishory A, Molnar C, Koola J, Li X, Kozel FA, Myrick H, et al. The Maximum-likelihood Strategy for Determining Transcranial Magnetic Stimulation Motor Threshold, Using Parameter Estimation by Sequential Testing Is Faster Than Conventional Methods With Similar Precision. J ECT. 2004;20:160–5. 15343000 10.1097/00124509-200409000-00007

[32] Schmitz A, Grillon C. Assessing fear and anxiety in humans using the threat of predictable and unpredictable aversive events (the NPU-threat test). Nat Protoc. 2012;7:527–32. 22362158 10.1038/nprot.2012.001 PMC3446242

[33] Blumenthal TD, Cuthbert BN, Filion DL, Hackley S, Lipp OV, van Boxtel A. Committee report: Guidelines for human startle eyeblink electromyographic studies. Psychophysiology. 2005;42:1–15. 15720576 10.1111/j.1469-8986.2005.00271.x

[34] Cox RW. AFNI: software for analysis and visualization of functional magnetic resonance neuroimages. Comput Biomed Res. 1996;29:162–73. 8812068 10.1006/cbmr.1996.0014

[35] Thielscher A, Antunes A, Saturnino GB. Field modeling for transcranial magnetic stimulation: A useful tool to understand the physiological effects of TMS? Proc. Annu. Int. Conf. IEEE Eng. Med. Biol. Soc. 2015;222–5, 10.1109/EMBC.2015.7318340. 10.1109/EMBC.2015.7318340 26736240

[36] Thielscher A, Opitz A, Windhoff M. Impact of the gyral geometry on the electric field induced by transcranial magnetic stimulation. NeuroImage. 2011;54:234–43. 20682353 10.1016/j.neuroimage.2010.07.061

[37] Balderston NL, Roberts C, Beydler EM, Deng Z-D, Radman T, Luber BM, et al. A generalized workflow for conducting electric field–optimized, fMRI-guided, transcranial magnetic stimulation. Nat Protoc. 2020;15:3595–614. 33005039 10.1038/s41596-020-0387-4 PMC8123368

[38] Borckardt JJ, Nahas Z, Koola J, George MS. Estimating Resting Motor Thresholds in Transcranial Magnetic Stimulation Research and Practice. J ECT. 2006;22:169–75. 16957531 10.1097/01.yct.0000235923.52741.72

[39] Forman SD, Cohen JD, Fitzgerald M, Eddy WF, Mintun MA, Noll DC. Improved assessment of significant activation in functional magnetic resonance imaging (fMRI): use of a cluster-size threshold. Magn Reson Med 1995;33:636–47. 7596267 10.1002/mrm.1910330508

[40] Cox RW, Chen G, Glen DR, Reynolds RC, Taylor PA. FMRI Clustering in AFNI: False-Positive Rates Redux. Brain Connect. 2017;7:152–71. 28398812 10.1089/brain.2016.0475 PMC5399747

[41] Barbey AK, Koenigs M, Grafman J. Dorsolateral prefrontal contributions to human working memory. Cortex J Devoted Study Nerv Syst Behav 2013;49:1195–205. 10.1016/j.cortex.2012.05.022 PMC3495093 22789779

[42] Altamura M, Elvevåg B, Blasi G, Bertolino A, Callicott JH, Weinberger DR, et al. Dissociating the effects of Sternberg working memory demands in prefrontal cortex. Psychiatry Res. 2007;154:103–14. 17292590 10.1016/j.pscychresns.2006.08.002

[43] Curtis CE, D’Esposito M. Persistent activity in the prefrontal cortex during working memory. Trends Cogn Sci 2003;7:415–23. 12963473 10.1016/s1364-6613(03)00197-9

[44] Geier CF, Garver KE, Luna B. Circuitry underlying temporally extended spatial working memory. NeuroImage. 2007;35:904–15. 17292627 10.1016/j.neuroimage.2006.12.022 PMC4397654

[45] Feredoes E, Heinen K, Weiskopf N, Ruff C, Driver J. Causal evidence for frontal involvement in memory target maintenance by posterior brain areas during distracter interference of visual working memory. Proc Natl Acad Sci 2011;108:17510–5. 21987824 10.1073/pnas.1106439108 PMC3198359

[46] Böcker KBE, Baas JMP, Leon Kenemans J, Verbaten MN. Differences in startle modulation during instructed threat and selective attention. Biol Psychol 2004;67:343–58. 15294391 10.1016/j.biopsycho.2004.01.001

[47] Grillon C, Ameli R. Effects of threat of shock, shock electrode placement and darkness on startle. Int J Psychophysiol 1998;28:223–31. 9545658 10.1016/s0167-8760(97)00072-x

[48] Alpers GW, Abelson JL, Wilhelm FH, Roth WT. Salivary cortisol response during exposure treatment in driving phobics. Psychosom Med 2003;65:679–87. 12883122 10.1097/01.psy.0000073872.85623.0c

[49] Braune S, Albus M, Frohler M, Hohn T, Scheibe G. Psychophysiological and Biochemical-Changes in Patients with Panic Attacks in a Defined Situational Arousal. Eur Arch Psychiatry Clin Neurosci 1994;244:86–92. 7948059 10.1007/BF02193524

[50] Charney DS, Heninger GR, Breier A. Noradrenergic function in panic anxiety. Effects of yohimbine in healthy subjects and patients with agoraphobia and panic disorder. Arch Gen Psychiatry. 1984;41:751–63. 6742977 10.1001/archpsyc.1984.01790190025003

[51] Coplan JD, Goetz R, Klein DF, Papp LA, Fyer AJ, Liebowitz MR, et al. Plasma cortisol concentrations preceding lactate-induced panic. Psychological, biochemical, and physiological correlates. Arch Gen Psychiatry. 1998;55:130–3. 9477926 10.1001/archpsyc.55.2.130

[52] Hoehn T, Braune S, Scheibe G, Albus M. Physiological, biochemical and subjective parameters in anxiety patients with panic disorder during stress exposure as compared with healthy controls. Eur Arch Psychiatry Clin Neurosci. 1997;247:264–74. 9444496 10.1007/BF02900305

[53] Parente AC, Garcia-Leal C, Del-Ben CM, Guimarães FS, Graeff FG. Subjective and neurovegetative changes in healthy volunteers and panic patients performing simulated public speaking. Eur Neuropsychopharmacol. 2005;15:663–71. 15961294 10.1016/j.euroneuro.2005.05.002

[54] Feeser M, Prehn K, Kazzer P, Mungee A, Bajbouj M. Transcranial direct current stimulation enhances cognitive control during emotion regulation. Brain Stimul. 2014;7:105–12. 10.1016/j.brs.2013.08.006. 10.1016/j.brs.2013.08.006 24095257

[55] Kroes MCW, Dunsmoor JE, Hakimi M, Oosterwaal S; NYU PROSPEC collaboration; Meager MR, et al. Patients with dorsolateral prefrontal cortex lesions are capable of discriminatory threat learning but appear impaired in cognitive regulation of subjective fear. Soc Cogn Affect Neurosci. 2019;14:601–12. 10.1093/scan/nsz039. 10.1093/scan/nsz039 PMC6688449 31119295

[56] Mayberg HS, Liotti M, Brannan SK, McGinnis S, Mahurin RK, Jerabek PA, et al. Reciprocal Limbic-Cortical Function and Negative Mood: Converging PET Findings in Depression and Normal Sadness. Am J Psychiatry. 1999;156:675–82. 10327898 10.1176/ajp.156.5.675

[57] Damasio AR, Grabowski TJ, Bechara A, Damasio H, Ponto LLB, Parvizi J, et al. Subcortical and cortical brain activity during the feeling of self-generated emotions. Nat Neurosci. 2000;3:1049–56. 11017179 10.1038/79871

[58] Davidson RJ, Irwin W. The functional neuroanatomy of emotion and affective style. Trends Cogn Sci. 1999;3:11–21. 10234222 10.1016/s1364-6613(98)01265-0

[59] Mantovani A, Aly M, Dagan Y, Allart A, Lisanby SH. Randomized sham controlled trial of repetitive transcranial magnetic stimulation to the dorsolateral prefrontal cortex for the treatment of panic disorder with comorbid major depression. J Affect Disord. 2013;144:153–9. 22858212 10.1016/j.jad.2012.05.038

[60] Diefenbach GJ, Bragdon LB, Zertuche L, Hyatt CJ, Hallion LS, Tolin DF, et al. Repetitive transcranial magnetic stimulation for generalised anxiety disorder: A pilot randomised, double-blind, sham-controlled trial. Br J Psychiatry. 2016;209:222–8. 27198484 10.1192/bjp.bp.115.168203

[61] White D, Tavakoli S. Repetitive transcranial magnetic stimulation for treatment of major depressive disorder with comorbid generalized anxiety disorder. Ann Clin Psychiatry. 2015;27:192–6. 26247218

[62] Caulfield KA, Stern AP. Therapeutic High-Frequency Repetitive Transcranial Magnetic Stimulation Concurrently Improves Mood and Anxiety in Patients Using Benzodiazepines. Neuromodulation. 2020;23:380–3. 31368628 10.1111/ner.13024

[63] Pell GS, Harmelech T, Zibman S, Roth Y, Tendler A, Zangen A. Efficacy of Deep TMS with the H1 Coil for Anxious Depression. J Clin Med. 2022;11:1015. 35207288 10.3390/jcm11041015 PMC8879826

[64] Dilkov D, Hawken ER, Kaludiev E, Milev R. Repetitive transcranial magnetic stimulation of the right dorsal lateral prefrontal cortex in the treatment of generalized anxiety disorder: A randomized, double-blind sham controlled clinical trial. Prog Neuropsychopharmacol Biol Psychiatry. 2017;78:61–5. 28533148 10.1016/j.pnpbp.2017.05.018

[65] Boggio PS, Rocha M, Oliveira MO, Fecteau S, Cohen RB, Campanhã C, et al. Noninvasive brain stimulation with high-frequency and low-intensity repetitive transcranial magnetic stimulation treatment for posttraumatic stress disorder. J Clin Psychiatry. 2010;71:992–9. 20051219 10.4088/JCP.08m04638blu PMC3260527

[66] Wilkes S, Ona C, Yang M, Liu P, Benton A, Lustik M, et al. Impacts of rTMS on Refractory Depression and Comorbid PTSD Symptoms at a Military Treatment Facility. Mil Med. 2020;185:e1420–e1427. 32617580 10.1093/milmed/usaa148

[67] van 't Wout-Frank M, Shea MT, Sorensen DO, Faucher CR, Greenberg BD, Philip NS. A Secondary Analysis on Effects of Theta Burst Transcranial Magnetic Stimulation to Reduce Anger in Veterans With Posttraumatic Stress Disorder. Neuromodulation. 2021;24:870–8. 32945055 10.1111/ner.13256 PMC8453662

[68] Petrosino NJ, Wout-Frank MV’T, Aiken E, Swearingen HR, Barredo J, Zandvakili A, et al. One-year clinical outcomes following theta burst stimulation for post-traumatic stress disorder. Neuropsychopharmacology. 2020;45:940–6. 31794974 10.1038/s41386-019-0584-4 PMC7162862

[69] Philip NS, Barredo J, Aiken E, Larson V, Jones RN, Tracie Shea M, et al. Theta-burst transcranial magnetic stimulation for posttraumatic stress disorder. Am J Psychiatry. 2019;176:939–48. 31230462 10.1176/appi.ajp.2019.18101160 PMC6824981

[70] LeDoux JE, Pine DS. Using Neuroscience to Help Understand Fear and Anxiety: A Two-System Framework. Am J Psychiatry. 2016;173:1083–93. 27609244 10.1176/appi.ajp.2016.16030353

[71] Hutton TM, Aaronson ST, Carpenter LL, Pages K, West WS, Kraemer C, et al. The Anxiolytic and Antidepressant Effects of Transcranial Magnetic Stimulation in Patients With Anxious Depression. J Clin Psychiatry. 2023;84. 10.4088/JCP.22m14571 36630648

[72] White LK, Makhoul W, Teferi M, Sheline YI, Balderston NL. The role of dlPFC laterality in the expression and regulation of anxiety. Neuropharmacology. 2023;224:109355. 36442650 10.1016/j.neuropharm.2022.109355 PMC9790039

[73] Nagel BJ, Herting MM, Maxwell EC, Bruno R, Fair D. Hemispheric lateralization of verbal and spatial working memory during adolescence. Brain Cogn. 2013;82:58–68. 23511846 10.1016/j.bandc.2013.02.007 PMC3652620

[74] Sheffer CE, Mennemeier MS, Landes RD, Dornhoffer J, Kimbrell T, Bickel WK, et al. Focal electrical stimulation as an effective sham control for active rTMS and biofeedback treatments. Appl Psychophysiol Biofeedback. 2013;38:171–6. 23702828 10.1007/s10484-013-9221-x PMC3882003

[75] González-Trejo E, StraussDJ, and Schwerdtfeger K, Transcranial magnetic stimulation (TMS): Development of an alternative placebo system, 2011 5th International IEEE/EMBS Conference on Neural Engineering, Cancun, Mexico, 2011, pp. 580–583, 10.1109/NER.2011.5910615.

[76] Rossi S, Ferro M, Cincotta M, Ulivelli M, Bartalini S, Miniussi C, et al. A real electro-magnetic placebo (REMP) device for sham transcranial magnetic stimulation (TMS). Clin Neurophysiol. 2007;118:709–16. 17188568 10.1016/j.clinph.2006.11.005

[77] Borckardt JJ, Walker J, Branham RK, Rydin-Gray S, Hunter C, Beeson H, et al. Development and evaluation of a portable sham transcranial magnetic stimulation system. Brain Stimulat. 2008;1:52–9. 10.1016/j.brs.2007.09.003 PMC2677803 19424444

[78] Corp DT, Bereznicki HGK, Clark GM, Youssef GJ, Fried PJ, Jannati A, et al. Large-scale analysis of interindividual variability in theta-burst stimulation data: Results from the ‘Big TMS Data Collaboration’. Brain Stimulat. 2020;13:1476–88. 10.1016/j.brs.2020.07.018 PMC7494610 32758665

