# Supplementary figures and images for: Intermittent theta-burst stimulation to the right dorsolateral prefrontal cortex may increase potentiated startle in healthy individuals
Source: Neuropsychopharmacology. 2024 May 13;49(10):1619–29. doi: 10.1038/s41386-024-01871-w (PMC11319663; doi:10.1038/s41386-024-01871-w)

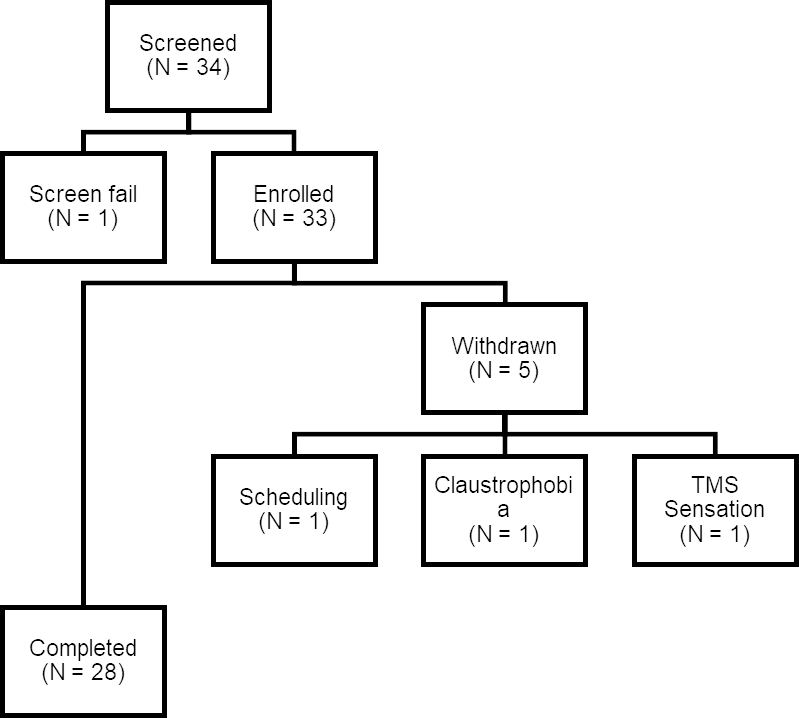

Supplement: Supplementary file 1 — Consort Diagram [file 41386_2024_1871_MOESM1_ESM.png]
